# Risk factors for cardiovascular disease mortality in patients with myelodysplastic syndromes: A nationwide, registry‐based cohort study

**DOI:** 10.1002/jha2.30

**Published:** 2020-07-01

**Authors:** Konstantinos Liapis, Georgios Vrachiolias, Vasileios Papadopoulos, Alexandra Kourakli, Athanasios G. Galanopoulos, Menelaos Papoutselis, Sotirios G. Papageorgiou, Panagiotis T. Diamantopoulos, Vassiliki Pappa, Nora‐Athina Viniou, Theodoros P. Vassilakopoulos, Eleftheria Hatzimichael, Eleni Bouronikou, Maria Ximeri, Charalambos Pontikoglou, Panayiotis Panayiotidis, Stamatis Karakatsanis, Anna Vardi, Argiris Symeonidis, Ioannis Kotsianidis

**Affiliations:** ^1^ Department of Hematology Democritus University of Thrace Medical School Alexandroupolis Greece; ^2^ Department of Internal Medicine University Hospital of Patras Rio Greece; ^3^ Department of Clinical Hematology Georgios Gennimatas Hospital Athens Greece; ^4^ Second Department of Internal Medicine Attikon University General Hospital Athens Greece; ^5^ First Department of Internal Medicine National and Kapodistrian University of Athens Athens Greece; ^6^ Department of Hematology Laikon General Hospital National and Kapodistrian University of Athens Athens Greece; ^7^ Department of Hematology University Hospital of Ioannina Ioannina Greece; ^8^ Department of Hematology University Hospital of Larissa Larissa Greece; ^9^ Department of Hematology University General Hospital of Heraklion Heraklion Greece; ^10^ First Propaedeutic Department of Internal Medicine National and Kapodistrian University of Athens Athens Greece; ^11^ Department of Hematology Sotiria General Hospital Athens Greece; ^12^ Department of Hematology and Stem Cell Transplantation Georgios Papanicolaou General Hospital Thessaloniki Greece

**Keywords:** cardiovascular disease, coronary heart disease, death, erythropoiesis‐stimulating agents, mortality, myelodysplastic syndromes

## Abstract

Cardiovascular disease (CVD) emerges as a major cause of death in patients with myelodysplastic syndrome (MDS), but predictors of fatal CVD and the effect of MDS‐specific treatments on CVD mortality remain largely unknown. In an analysis involving 831 patients with MDS with known causes of death, we noted an independent association of lower risk MDS, age >70 years, pre‐existing CVD, and treatment with erythropoiesis‐stimulating agents with a higher risk of death from CVD. If externally validated, these simple risk factors could increase clinicians’ awareness toward CVD complications and guide early introduction of intensive monitoring and preventive interventions in MDS patients.

## 1 INTRODUCTION

Increasing evidence exists of an overlap in the risk factors between the development of cardiovascular disease (CVD) and cancer [1], and many individuals diagnosed with cancer have underlying cardiovascular disease risk factors, such as advancing age, hypertension, tobacco use, and diabetes. Myelodysplastic syndromes (MDS) are a heterogeneous group of clonal stem cell‐derived disorders characterized by cytopenias, ineffective hemopoiesis, and a substantial risk of progression to acute myeloid leukemia. MDS typically affect elderly individuals with several comorbidities, which may influence treatment decisions and clinical outcomes, independently of the International Prognostic Scoring System (IPSS) [2].

Recent data argue for an intrinsic tendency of MDS toward the development of CVD. In 2017, Jaiswal and coworkers described nearly a doubling in the risk of coronary heart disease in persons with clonal hematopoiesis of indeterminate potential (CHIP), a precursor of MDS [3]. In the same year, Brunner and colleagues found that the burden of cardiovascular deaths was especially noteworthy in lower risk MDS, as compared with higher risk disease [4]. However, findings from the above studies seem inconsistent with the study by Adrianzen Herrera and coworkers who reported increased risk of incident CVD in patients with higher risk and transfusion‐dependent MDS [5], whereas no study has investigated the effect of MDS‐specific treatments on CVD morbidity and mortality.

The underlying pathophysiology of the increased prevalence of CVD in CHIP and MDS is not clear, but systemic inflammation and recurrent somatic mutations may contribute to accelerated atherogenesis [6, 7]. However, the biggest challenges at the moment are to identify simple, MDS‐specific predictors for CVD morbidity and mortality and understand the role of MDS treatments within this complex picture. The latter is undoubtedly a key health issue. In this study, we aimed to detect commonly used clinical and laboratory parameters that would help hematologists to recognize patients with MDS at increased risk of death from CVD.

## 2 PATIENTS AND METHODS

The Hellenic National Registry of Myelodysplastic and Hypoplastic Syndromes has retrospectively collected data from 2972 patients with MDS, myelodysplastic/myeloproliferative neoplasms, and low‐blast‐count acute myeloid leukemia (AML) diagnosed between December 1985 and June 2016. In the current study, we performed a subanalysis of 831 patients with known chief, not contributory, causes of death (COD). The data cutoff date for the analysis was 7 July 2016. Response to erythropoiesis‐stimulating agents (ESAs) was evaluated using the International Working Group criteria [8]. Red cell transfusion dependency was defined as having at least one red cell transfusion every 8 weeks over a period of 4 months, according to the WHO classification‐based prognostic scoring system [9]. Comorbidity and multimorbidity were defined according to the MDS‐specific comorbidity index (MDS‐CI) [10]. Data on hemoglobin concentration, transfusion dependency, and serum ferritin were recorded at diagnosis prior to any intervention. In line with recent studies, CVD in our patient cohort encompassed (a) coronary artery disease (myocardial infarction, ischemic cardiomyopathy, or history of percutaneous coronary intervention or coronary artery bypass graft), (b) cerebrovascular events (stroke or transient ischemic attack), (c) peripheral artery disease, and (d) thoracic or abdominal aortic aneurysms [4, 11]. We further assessed the Endothelial Activation and Stress Index (EASIX), a novel marker for endothelial dysfunction in MDS [12], calculated as lactate dehydrogenase (LDH; ΙU/L) × creatinine (mg/dL)/platelet count (× 10^9^/L). Survival analysis was performed using a Kaplan‐Meier estimate. Overall survival (OS) was defined as the time from MDS diagnosis to last follow‐up or death from any cause and leukemia‐free survival (LFS) as the time from MDS diagnosis to leukemic progression or death. Details of statistical analysis are included in the Appendix in the Supporting Information.

## 3 RESULTS

Table 1 lists patients’ baseline characteristics with pairwise comparisons for all the prognostic variables considered. CVD as a COD was registered in 108 patients (CVD death group), whereas 723 patients died from other causes (non‐CVD death group). After a median follow‐up of 50.0 months (range 46.3‐53.7), the median OS and LFS of patients in CVD death group were 30.0 months (95% CI, 23.0‐37.0) and 29.0 months (95% CI, 21.9‐36.1), respectively, whereas the median OS and LFS of patients in the non‐CVD death group were 20.0 months (95% CI, 18.2‐21.8) and 17.0 months (95% CI, 15.4‐18.6), respectively (Figure S1). Eleven out of 107 patients in the CVD death group (10.3%) and 403 of 683 (59.1%) in the non‐CVD death group developed AML (*P* < .001).

**TABLE 1:** Demographic, clinical, laboratory, and histologic findings and treatments of patients with myelodysplastic syndromes whose death was attributed to cardiovascular disease (CVD mortality) versus those who died from a noncardiovascular disease cause (non‐CVD mortality)

Parameters	CVD mortality (n = 108)	Non‐CVD mortality (n = 723)	*P*‐value
Demographics and clinical characteristics			
Age (years, median [range])	75.5 (55‐96)	73 (34‐93)	<.001
Sex			.379
Male	72/108 (66.7%)	512/723 (70.8%)	
Female	36/108 (33.3%)	211/723 (29.2%)	
ECOG PS			.125
0	29/105 (27.6%)	265/675 (39.3%)	
1	44/105 (41.9%)	267/675 (39.6%)	
2	26/105 (24.8%)	117/675 (17.3%)	
3	6/105 (5.7%)	25/675 (3.7%)	
4	0/105 (0.0%)	1/675 (0.1%)	
ND	3	48	
Risk factors for CVD^a^			<.001
Absence of risk factors for CVD	20/102 (19.6%)	267/667 (40%)	
1 risk factor for CVD present	19/102 (18.6%)	144/667 (21.6%)	
2 risk factors for CVD present	5/102 (4.9%)	62/667 (9.3%)	
3 risk factors for CVD present	0/102 (0.0%)	4/667 (0.6%)	
ND	6	56	
Pre‐existing CVD	58/102 (56.9%)	190/667 (28.5%)	<.001
Hepatic comorbidity^b^			.221
Yes	0	10/723 (1.4%)	
No	107/107 (100%)	714/723 (98.6%)	
ND	1	0	
Pulmonary comorbidity^b^			.075
Yes	20/107 (18.7%)	90/723 (12.4%)	
No	87/107 (81.3%)	633/723 (87.6%)	
ND	1	0	
Renal comorbidity^b^			.169
Yes	8/107 (7.5%)	32/723 (4.4%)	
No	99/107 (92.5%)	691/723 (95.6%)	
ND	1	0	
Solid tumor^b^			.048
Yes	4/107 (3.7%)	69/723 (9.5%)	
No	103/107 (96.3%)	654/723 (90.5%)	
ND	1	0	
MDS‐CI			<.001
1 (low risk)	42/99 (42.4%)	402/653 (61.6%)	
2 (intermediate risk)	46/99 (46.5%)	221/653 (33.8%)	
3‐4 (high risk)	11/99 (11.1%)	30/653 (4.6%)	
ND	9	70	
Laboratory findings			
Hemoglobin (g/dL, median [range])	9.0 (5.2‐13.6)	9.2 (4.0‐15.2)	.133
Absolute neutrophil count (× 10^9^/L, median [range])	5.3 (1.4‐12.3)	3.7 (0.5‐72.1)	.241
Platelet count (× 10^9^/L, median [range])	190 (10‐846)	105.5 (0.0‐1279)	<.001
Lactate dehydrogenase (U/L, median [range])	203 (96‐850)	233 (75‐1176)	.042
Serum ferritin (μg/L, median [range])	333 (16.0‐1282)	269.5 (1.6‐6755)	.685
β_2_‐Microglobulin (mg/L, median [range])	3 (1.0‐13.7)	1.83 (0.8‐17.8)	.735
eGFR (mL/min/1.73 m^2^, median [range])	61.1 (24.0‐104.1)	68.1 (11.2‐143)	.162
Log_2_(EASIX) score (median [range])	0.3 (‐1.4 – 3.3)	0.8 (‐1.6 – 5.8)	<.001
Histologic findings			
Peripheral blood blast count (%, median [range])	0.0 (0‐8)	0.0 (0‐17)	.001
Bone marrow blast count (%, median [range])	2.0 (0‐19)	6.0 (0‐19)	<.001
WHO 2008 classification			<.001
RCUD	35/105 (33.3%)	127/686 (18.5%)	
RCMD	19/105 (18.1%)	115/686 (16.8%)	
RAEB‐1	16/105 (15.2%)	180/686 (26.2%)	
RAEB‐2	12/105 (11.4%)	197/686 (28.7%)	
RARS	16/105 (15.2%)	21/686 (3.1%)	
RCMD‐RS	6/105 (5.7%)	29/686 (4.2%)	
del(5q) MDS	1/105 (1.0%)	9/686 (1.3%)	
MDS‐U	0/105 (0.0%)	3/686 (0.4%)	
CMML	0/105 (0.0%)	5/686 (0.7%)	
ND	3	37	
IPSS prognostic risk category			<.001
Low	47/100 (47%)	113/668 (16.9%)	
Intermediate‐1	36/100 (36%)	278/668 (41.6%)	
Intermediate‐2	13/100 (13%)	182/668 (27.2%)	
High	4/100 (4.0%)	95/668 (14.2%)	
ND	8	55	
Revised‐IPSS prognostic risk category			<.001
Very low	11/99 (11.1%)	49/666 (7.4%)	
Low	50/99 (50.5%)	172/666 (25.8%)	
Intermediate	16/99 (16.2%)	155/666 (23.3%)	
High	18/99 (18.2%)	159/666 (23.9%)	
Very high	4/99 (4.0%)	131/666 (19.7%)	
ND	9	57	
Treatments			
Red cell transfusion dependency^c^			.677
Yes	57/106 (53.8%)	396/708 (55.9%)	
No	49/106 (46.2%)	312/708 (44.1%)	
ND	2	15	
Iron‐chelating agents			.787
Yes	8/57 (14.0%)	46/361 (12.7%)	
No	49/57 (86.0%)	315/361 (87.3%)	
ND	51	362	
ESA use^d^			.001
Yes	83/100 (83%)	436/657 (66.3%)	
No	17/100 (17%)	216/657 (33.7%)	
ND	8	71	
Response to ESAs^e^			.594
Yes	26/72 (36.1%)	117/356 (32.9%)	
No	46/72 (63.9%)	239/356 (67.1%)	
ND	11	80	
Other first‐line treatments			.501
Hypomethylating agents	7/100 (7%)	123/652 (18.9%)	
Lenalidomide	2/100 (2%)	9/652 (1.4%)	
Low‐dose cytarabine	1/100 (1%)	12/652 (1.8%)	
Intensive chemotherapy^f^	1/100 (1%)	20/652 (2.8%)	
Other drugs^g^	6/100 (5%)	57/652 (4.4%)	
ΝD	8	71	

Abbreviations: CMML, chronic myelomonocytic leukemia; CVD, cardiovascular disease; del(5q) MDS, myelodysplastic syndrome associated with isolated del(5q); EASIX, endothelial activation and stress index; ECOG PS, Eastern Cooperative Oncology Group performance status; eGFR, estimated glomerular filtration rate; ESAs, erythropoiesis‐stimulating agents; IPSS, International Prognostic Scoring System; MDS‐CI, myelodysplastic syndrome‐specific comorbidity index; MDS‐U, myelodysplastic syndrome unclassified; ND, not determined; RAEB‐1, refractory anemia with excess blasts 1; RAEB‐2, refractory anemia with excess blasts 2; RARS, refractory anemia with ring sideroblasts; RCMD, refractory cytopenia with multilineage dysplasia; RCMD‐RS, refractory cytopenia with multilineage dysplasia and ring sideroblasts; RCUD, refractory cytopenia with unilineage dysplasia (refractory anemia; refractory neutropenia; refractory thrombocytopenia); WHO, World Health Organization.

a Risk factor data were available for hypertension, dyslipidemia, and diabetes.

b According to the myelodysplastic syndrome‐specific comorbidity index (MDS‐CI).

c Red cell transfusion dependence was defined as having at least one red cell transfusion every 8 weeks over a period of 4 months, according to the WHO‐based prognostic scoring system (WPSS) [9].

d High‐dose erythropoiesis‐stimulating agents, including recombinant erythropoietin or darbepoetin alfa, with or without granulocyte colony‐stimulating factor (G‐CSF).

e Response to erythropoiesis‐stimulating agents (ESAs) was assessed according to the International Working Group (IWG) 2006 criteria.

f Intensive chemotherapy regimens included standard cytarabine and idarubicin or mitoxantrone combinations.

g Other drugs, such as antithymocyte globulins, with or without ciclosporin, androgens (danazol), and eltrombopag.

**FIGURE 1:**
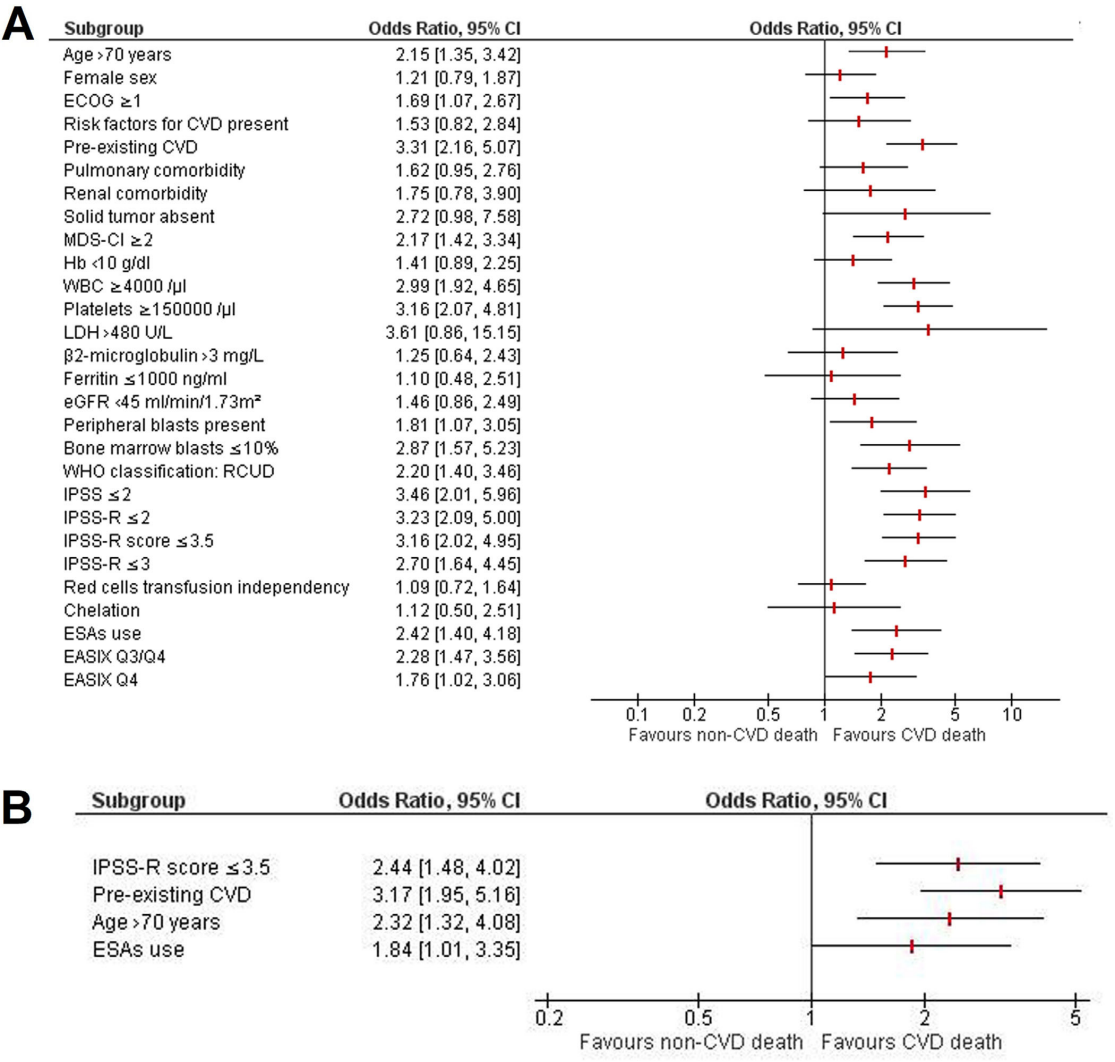
Odds ratio (OR) and 95% confidence interval (CI) for cardiovascular disease (CVD) mortality in patients with myelodysplastic syndromes. ORs and CIs were determined with binary regression analyses. A, Forest plot of univariate OR (95% CI). B, Forest plot of multivariate OR (95% CI) showing the four variables associated with statistical significance (*P* < .05) in our multivariate logistic regression model Abbreviations: EASIX, endothelial activation and stress index; ECOG, Eastern Cooperative Oncology Group performance status; eGFR, estimated glomerular filtration rate; ESAs, erythropoiesis‐stimulating agents; Hb, hemoglobin; IPSS, International Prognostic Scoring System; IPSS‐R, Revised International Prognostic Scoring System; LDH, lactate dehydrogenase levels; MDS‐CI, myelodysplastic syndrome‐specific comorbidity index; Q3, third quartile; Q4, fourth quartile; RCUD, refractory cytopenia with unilineage dysplasia (refractory anemia; refractory neutropenia; refractory thrombocytopenia); WBC, white blood cell count; WHO, World Health Organization

Univariate analysis at diagnosis (Table 1 and Figure 1A) found no differences between the two groups in performance status, liver, pulmonary and renal comorbidity, gender, and red blood cell (RBC) transfusion dependency. By contrast, patients in the CVD death group were older and had higher prevalence of pre‐existing CVD and lower risk MDS. MDS‐CI was also significantly increased among patients in the CVD death group as compared to patients in the non‐CVD death group, most likely because cardiac comorbidity is the highest top‐scoring parameter in the calculation of MDS‐CI. Previously diagnosed solid tumors were slightly increased in the non‐CVD death group, but the low number of patients with this comorbidity in the CVD‐death group (n = 4) precludes any definite conclusion. Regarding hematological and biochemical parameters, the neutrophil count, hemoglobin concentration, serum ferritin, and β2‐microglobulin levels were similar between the two groups, whereas lower platelet count, higher peripheral blood and bone marrow blast percentages, and higher LDH levels were found in the non‐CVD death group. We noted significantly higher EASIX values in the non‐CVD death group but, because the calculation of EASIX depends of LDH and platelet count, these results may simply reflect the higher risk disease status of patients in this group.

Of note, more patients in the CVD death group were treated with ESAs with or without granulocyte colony‐stimulating factor (G‐CSF) than the non‐CVD death group (*P* < .001), although responses to ESAs ± G‐CSF were comparable between the two groups (*P* = .594). No difference in other first‐line regimens was observed between the two groups (*P* = .501), including chelating agents (*P* = .787).

In multivariate analysis, only four parameters correlated independently with CVD death: lower risk MDS, namely, an IPSS‐R score ≤3.5 (odds ratio [OR] = 2.44; 95% CI, 1.48‐4.03; *P* < .001), pre‐existing CVD (OR = 3.17; 95% CI, 1.95‐5.15; *P* < .001), age >70 years (OR = 2.32; 95% CI, 1.32‐4.08; *P* = .004), and exposure to ESAs (OR = 1.84; 95% CI, 1.01‐3.36; *P* = .047), as shown in Figure 1B. Because both OS and LFS were longer in the CVD death group, it could be argued that the longer survival and lower AML transformation rate of these patients skews the cause of death toward CVD, irrespective of the type of treatment used. To achieve increased statistical power and better quality assessment, we further developed a univariate general linear model over imputed data (total number of imputations = 5) using CVD death as the dependent variable and pre‐existing CVD, ESA use, age >70 years, and IPSS‐R score ≥3.5 as independent variables. The results confirmed that pre‐existing CVD (*P* = .007), ESA use (*P* = .008), age >70 years (*P* = .011), and IPSS‐R ≥3.5 (*P* = .015) presented a statistically significant between‐subject effect and, thus, were all independently associated with CVD death.

## 4 DISCUSSION

In this large, nationwide cohort of MDS patients with known outcomes, we evaluated the effect of numerous clinical and laboratory variables on CVD mortality. Our findings seemingly challenge some of the basic results in the study by Adrianzen Herrera and co‐workers who reported an increased risk for CVD events in higher risk, transfusion‐dependent patients with a Charlson Comorbidity Index (CCI) >1 [5]. We found no association of cardiovascular death with transfusion dependence and a strong association with lower risk disease according to IPSS‐R, whereas neither single comorbidity nor the MDS‐CI was independently associated with cardiovascular mortality in our analysis. This is likely to be attributable to notable differences in methodological approaches: first, the aforementioned study evaluated the occurrence of incident CVD, whereas we focused only on cardiovascular death. Second, transfusion dependency was defined as two or more transfusions within 60 days of diagnosis, whereas we used the criterion of at least one RBC transfusion every 8 weeks over a period of 4 months [9]. Transfusion intensity has been previously linked with death from CVD [9], therefore the more rigid definition of transfusion dependency used by Adrianzen Herrera and colleagues may potentially represent a cause for the discrepant results. Nevertheless, hemoglobin levels, a more objective risk factor for CVD complications in MDS [9], were similar in both patient groups of our cohort, whereas our multivariate analysis included several other potentially important confounders for CVD risk, namely, serum ferritin, estimated glomerular filtration rate, ESA treatment, pre‐existing CVD, and MDS‐CI.

We also noted that ESA use correlated independently with increased cardiovascular deaths. Although in a meta‐analysis of 57 studies including 9353 patients with (mostly) solid tumors ESA use was associated with a 1.67‐fold increased risk of venous plus arterial thrombosis [13], only one study has investigated the effect of ESAs on cardiovascular events in MDS. In this cohort of 2114 MDS patients, a 1.6‐fold and twofold increased risk for myocardial infarction and stroke, respectively, was associated with ESA treatment, but no information on the concomitant use of G‐CSF was provided and this effect was not controlled for anemia, an important confounder [14]. The almost identical 1.92‐fold increment seen in our study—adjusted, among others, for the degree of anemia and renal function—suggests that ESA ± G‐CSF use may be involved in CVD development in MDS patients. However, taking into account the vast complexity of interactions among treatments, MDS and atherothrombosis, even independent correlations may not necessary imply causality. Certainly, our results do not negate the possible favorable effect of ESAs on hemoglobin level and disease course; instead, they suggest that clinicians may need to exercise additional caution when managing elderly patients with lower risk MDS treated with ESAs, especially those with a known baseline history and/or risk factors for CVD.

Furthermore, our analysis is subject to the inherent limitations of retrospective studies. We could not consider changes in the risk factor variables during follow‐up or the effects of treating risk factors, and we could not analyze the effects of smoking due to the high number of missing data. Previous works also lack data on smoking history, suggesting a general lack of awareness of tobacco‐related harms in patients with MDS [4, 5]. Nevertheless, the large patient cohort, nationwide coverage, two‐group pragmatic approach focusing only on fatal CVD, and the inclusion of clinically essential parameters, including first line therapies, represent‐ key strengths of our study.

In summary, our study expands on what is known about CVD deaths in MDS. From the practical point of view, if validated in independent cohorts, the potential risk factors of older age, revised‐IPSS score value ≤3.5, pre‐existing CVD, and ESA use could help in early identification of patients at risk for CVD events and guide preventive strategies. However, molecular profiling and prospective collection of data on therapeutic interventions and additional risk factors for CVD, such as lifestyle and family history, are needed to utterly define MDS‐specific predictors of CVD morbidity and mortality.

## AUTHOR CONTRIBUTIONS

IK conceived the idea for this study and designed the study. V Papadopoulos contributed to the concept of the study. IK, V Papadopoulos, KL, and GV collected, analyzed, and interpreted the data, reviewed the literature, and wrote the manuscript. AK, AGG, MP, SGP, PTD, V Pappa, NAV, TPV, EH, EB, MX, CP, PP, SK, AV, and AS collected the data. All authors reviewed and approved the final version of the manuscript.

## ORIGINALITY

This manuscript contains original material that has not been published or submitted previously to another journal. All authors agree to the submission of this manuscript to the British Journal of Hematology.

## CONFLICT OF INTERESTS

IK, NAV, AS, EH, and V Pappa have received research funding from Celgene Corporation. IK, SGP, TPV, AGG, EH, PP, AK, AS, V Pappa, and NAV have received honoraria from Genesis Pharma Hellas S.A.

## Supporting information

Supporting Information
