# Supplementary material for: Risk factors for cardiovascular disease mortality in patients with myelodysplastic syndromes: A nationwide, registry‐based cohort study
Source: EJHaem. 2020 Jul 1;1(1):255–61. doi: 10.1002/jha2.30 (PMC9176014; doi:10.1002/jha2.30)

**REVISED MANUSCRIPT: ejhaem-2020-05-0051.R1**

**SUPPLEMENTARY APPENDIX**

**Supplement to:**

Short Report

**Risk factors for fatal cardiovascular disease in patients with myelodysplastic syndromes: a nationwide, registry-based cohort study**

Konstantinos Liapis, Georgios Vrachiolias, Vasileios Papadopoulos, Alexandra Kourakli, Athanasios G. Galanopoulos, Menelaos Papoutselis, Sotirios G. Papageorgiou, Panagiotis T. Diamantopoulos, Vasiliki Pappa, Nora-Athina Viniou, Theodoros P. Vassilakopoulos, Eleftheria Hatzimichael, Eleni Bouronikou, Maria Ximeri, Charalambos Pontikoglou, Panayiotis Panayiotidis, Stamatis Karakatsanis, Anna Vardi, Argiris Symeonidis, Ioannis Kotsianidis

**Statistical methods**

Kaplan-Meier curves were used to depict survival data. Calculation of median follow-up time was based on the reverse Kaplan-Meier estimator method applied to the whole Registry. To explore the risk factors associated with cardiovascular-disease‒related death, univariate and multivariate analyses were used. Univariate comparisons were performed with the use of Pearson’s χ^2^ or a two-sample independent t-test in case of discrete and continuous variables respectively. Fisher’s exact test was preferred instead of Pearson’s χ2 in case that more than one cell had expected frequencies below 5. Τo check the homogeneity of variance, we used Levene’s test. Multivariate analysis of risk factors was performed with binary logistic regression; cardiovascular-disease‒related death was considered as a dependent variable and every parameter that was significantly correlated in univariate analysis (p ≤0.05) was treated as a potential independent parameter (the probability for stepwise entry and removal were set to 0.05 and 0.10 accordingly, the classification cutoff was set to 0.5, and the maximum number of iterations was set to 20). Outliers, defined as observations falling “far out” Tukey’s fences (<Q1 − 3*IQR or >Q3 + 3*IQR, where Q1=1^st^ quartile, Q3=3^rd^ quartile, and IQR=interquartile range) were excluded from our analysis. Data imputation was used for missing data when stated. All reported p values are two-sided. The level of statistical significance was set to p=0.05; values of 0.05≤p≤0.1 were considered as needed further evaluation. All numerical values are given with at least two significant digits.

Statistical analysis was performed with the use of IBM SPSS Statistics software, version 20.0, for Windows. MedCalc Statistical Software version 19.1 (MedCalc Software, Ostend, Belgium; 2019) was used for visualisation of survival curves, and Review Manager version 5.3 (RevMan, Copenhagen: The Nordic Cochrane Centre, The Cochrane Collaboration; 2014) was used to illustrate forest plots.

**Figure S1.** Kaplan–Meier curves of overall survival (left) and leukaemia-free survival (right) for patients with myelodysplastic syndromes according to the cause of death (cardiovascular mortality versus other mortality).


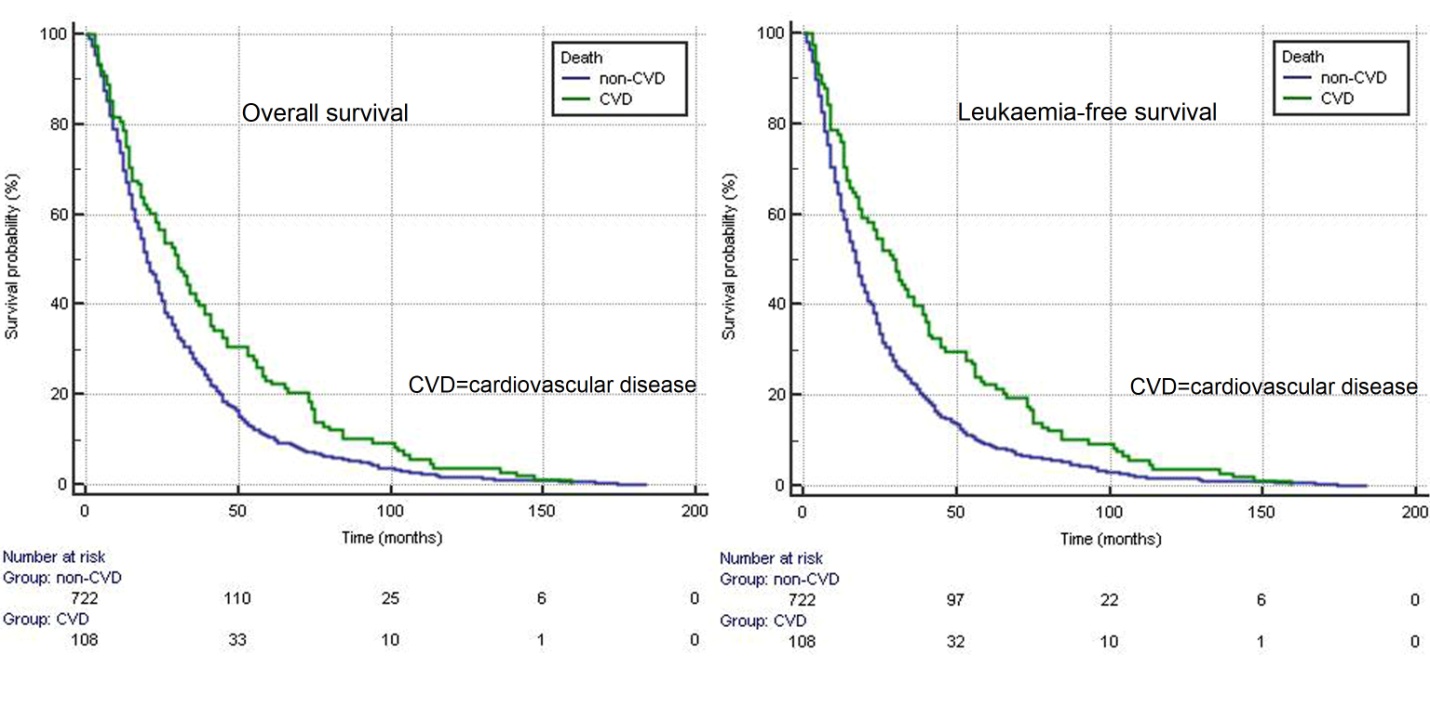

Supplement: Supplementary file 1 — Supporting Information [file JHA2-1-255-s001.docx]
